# Transcriptomic analysis of *Lacticaseibacillus paracasei* Zhang in transition to the viable but non-culturable state by RNA sequencing

**DOI:** 10.3389/fmicb.2023.1280350

**Published:** 2023-12-21

**Authors:** Qiuhua Bao, Xuebo Ma, Xiaoyu Bo, Jing Pang, Lixia Dai, Huiying Wang, Yongfu Chen, Lai-Yu Kwok

**Affiliations:** ^1^Key Laboratory of Dairy Biotechnology and Engineering, Ministry of Education, Inner Mongolia Agricultural University, Hohhot, China; ^2^Key Laboratory of Dairy Products Processing, Ministry of Agriculture and Rural Affairs, Inner Mongolia Agricultural University, Hohhot, China; ^3^Inner Mongolia Key Laboratory of Dairy Biotechnology and Engineering, Inner Mongolia Agricultural University, Hohhot, China

**Keywords:** *Lacticaseibacillus paracasei* Zhang, viable but non-culturable (VBNC), induction, high-throughput RNA sequencing, transcriptomics

## Abstract

**Background:**

Some bacteria enter the viable but non-culturable (VBNC) state to survive harsh environmental conditions and external stresses. This alters cell physiology and has implications for the food industry as some bacteria, such as lactobacilli, undergo similar changes during food processing.

**Methods:**

This study aimed to investigate the transcriptomic changes of a probiotic strain, *Lacticaseibacillus paracasei* Zhang (*L. paracasei* Zhang), upon transition to the VBNC state using high throughput RNA sequencing (RNA-seq).

**Results:**

Bacteria were inoculated into the de Man, Rogosa, and Sharpe medium and maintained at low temperature and pH to induce cell transition to the VBNC state. Cells were harvested for analysis at five stages of VBNC induction: 0, 3, 30, and 180 days after induction and 210 days when the cells entered the VBNC state. Our results showed that the expression of 2,617, 2,642, 2,577, 2,829, and 2,840 genes was altered at these five different stages. The function of differentially expressed genes (DEGs, compared to healthy cells collected at day 0) and their encoded pathways were analyzed by the Gene Ontology Consortium and the Kyoto Encyclopedia of Genes and Genomes pathway enrichment analyses. A total of 10 DEGs were identified in cells that entered the VBNC state: five continuously upregulated (LCAZH_0621, LCAZH_1986, LCAZH_2038, LCAZH_2040, and LCAZH_2174) and five continuously downregulated (LCAZH_0024, LCAZH_0210, LCAZH_0339, LCAZH_0621, and LCAZH_0754).

**Conclusions:**

This study proposes a molecular model of the VBNC mechanism in *L. paracasei* Zhang, highlighting that changes in cell metabolism improve substrate utilization efficiency, thereby enhancing bacterial survival under adverse conditions. These data may be useful for improving the survival of probiotics in industrial food processing.

## Introduction

Lactic acid bacteria are a group of non-spore-producing anaerobic or facultative anaerobic Gram-positive cocci or bacilli used to produce fermented foods (Reid, 2016). They produce lactic acid by fermenting sugars for energy. However, these bacteria face environmental stresses, such as cold and heat stress, osmotic pressure stress, and oxygen stress, during manufacturing and storage processes (Hor et al., 2018). Orally ingested probiotics are also exposed to acidic, bile salt, and digestive enzyme-rich environments during the gastrointestinal transit (Davis, 2014; Fonseca et al., 2019). Under harsh environmental conditions, certain lactic acid bacteria transit to the viable but non-culturable (VBNC) state to maximize their survival (Hood and Ness, 1982).

In 1982, Xu et al. (1982) first reported the VBNC state in two bacterial species, *Vibrio cholerae* and *Escherichia coli*, in estuarine and marine environments. The concept of the VBNC state of bacteria was formalized by Colwell et al. (1985). Two hypotheses have been proposed regarding its formation mechanism: One is a result of starvation and degradation of cytoplasm, leading to cell loss, weakness, and apoptosis, and the other is that bacteria in the VBNC state do not have a tendency to die but program responses to adapt to adverse environments for survival (Whitesides and Oliver, 1997; McDougald et al., 1998; Nyström, 2001; Ayrapetyan et al., 2018). After entering the VBNC state, bacterial cells maintain minimal metabolic activity, resembling endospore formation. Bacterial strains enter the VBNC state under specific environmental conditions regulated by physical factors (such as temperature, humidity, oxygen concentration, and light intensity), chemical factors (such as nutrients and harmful chemicals), and biological factors (Liu et al., 2016; Schottroff et al., 2018; Alvear-Daza et al., 2020; Li et al., 2020; Wasfi et al., 2020; Zhang et al., 2020). For example, *Staphylococcus aureus* was induced to enter the VBNC state after 18-day citric acid treatment at low temperatures (Bai et al., 2019). *Aeromonas hydrophila* was induced to enter the VBNC state under starvation conditions (Maalej et al., 2004). Both acidic and alkaline conditions can trigger the VBNC state in bacteria (Darcan et al., 2009; Capozzi et al., 2016).

RNA sequencing (RNA-seq) is a method used to detect global transcriptomic changes in single cells or cell populations at the single nucleotide level. It identifies unknown and rare transcripts, analyzes their structure and expression levels, and provides comprehensive transcriptional information, aiding in a better understanding of gene function (Wang et al., 2009). RNA-seq has revealed changes in bacterial physiology and metabolism in VBNC states, such as protein synthesis and transmembrane transport in low-temperature-induced VBNC *Escherichia coli* O157:H7 (Zhong and Zhao, 2019), substrate transport, metabolic processes, and enzyme activities in VBNC *Lactobacillus acetotolerans*, a beer spoilage species (Liu et al., 2016), and elevated expression of antioxidant genes in oxidative stress-induced VBNC *Escherichia coli* (Chen et al., 2018). Research on the VBNC state of food bacteria is limited, especially for probiotic dairy strains used as starter bacteria and functional foods. Since probiotics need to remain alive to benefit the host after ingestion, it would be of interest to understand their biology underlying the VBNC state and precisely quantify their viability. Conventional cultivation methods often underestimate the viability of VBNC bacteria (Foglia et al., 2020). It is also important to profile their global physiological changes to identify biomarkers for enumerating VBNC cells in probiotic products in the long run.

*Lacticaseibacillus paracasei* Zhang (*L. paracasei* Zhang) is a probiotic isolated from naturally fermented mare milk in Inner Mongolia. The whole genome of *L. paracasei* Zhang was assembled and analyzed; its genome contains 31 phosphotransferase systems (PTSs), reflecting a strong sugar utilization ability (Zhang et al., 2010). The strain has been extensively studied and has shown beneficial effects on the host. Phage infection of starter cultures is a potential problem in the industrial production of fermented milk as it could affect the fermentation process. The genome of *L. paracasei* Zhang encodes a functional bacteriophage exclusion system that protects the cells against phage and exogenous DNA invasion, making it a suitable strain for industrial use (Hui et al., 2019). Its genome also encodes stress resistance genes to combat external insults such as antibiotics and acid stress via modulating membrane-associated proteins and regulating various signaling pathways (Wu et al., 2011).

The environmental-induced VBNC state of *L. paracasei* Zhang has, however, not been extensively studied. Since *L. paracasei* Zhang is increasingly used in industrial fermented milk production, it is imperative to comprehend the formation mechanism and biology of its VBNC state. This study utilized low temperatures to induce *L. paracasei* Zhang into the VBNC state, and RNA-seq was used to monitor transcriptomic changes in bacterial gene expression during the induction. The differentially expressed genes (DEGs) identified between healthy cells and those collected at various stages of VBNC induction were annotated by the Gene Ontology (GO) Consortium and Kyoto Encyclopedia of Genes and Genomes (KEGG) database. Our findings offer valuable insights into the physiology of the VBNC state of a dairy probiotic, which is of interest to the fermented dairy industry for future food and health food production.

## Materials and methods

### Bacterial strains and culture conditions

The *L. paracasei* Zhang strain was preserved and provided by the Key Laboratory of Dairy Products and Biotechnology, Inner Mongolia Agricultural University. It was deposited in the China General Microbiological Culture Collection Center (deposit number: 1697). The pure culture was stored in an ampoule with skim milk at −80°C. The strain was activated and subcultured in the de Man, Rogosa and Sharpe (MRS) broth at 37°C until reaching the exponential phase (16–18 h; around 1.8 × 10^9^ CFU/mL) for subsequent experiments.

### Induction of VBNC state

To induce the VBNC state, exponential phase cultures of *L. paracasei* Zhang were inoculated (2%) into liquid MRS medium (pH 3.8) and left at 4°C.

### Cultivability and viability assays

All procedures were performed aseptically. The cell culture intended for VBNC induction was gently mixed. Then, a 0.5-mL sample was taken and serially diluted using a 10-fold gradient. The diluted samples were then plated on MRS agar, and the inoculated agar plates were incubated aerobically at 37°C for 48 h before colony counting. The total number of colonies was recorded (in CFU/mL). The experiment was performed in triplicate. The count of viable cells was considered to be zero only when the plate count results were consistently negative after repeating the assays three times in a row.

Bacterial viability was determined using a live/dead BacLight™ Bacterial Viability Kit (L-7012, Molecular Probes, Inc., Eugene, OR, USA) based on cell activity. Cell cultures were washed twice with sterile saline NaCl (0.85%) solution and diluted appropriately to ensure proper bacterial density for counting under a microscope. Cells were then stained with SYTO 9 and propidium iodide according to the manufacturer's instructions. In brief, a volume of 2 μL mixture (1.5 μL each of SYTO 9 and propidium iodide dissolved in 100 μL of dimethyl sulfoxide) was added to 50 μL of cell suspension. After 15 min of staining at 25°C, 2 μL of this suspension was transferred to a glass slide. The cells were observed and counted under a Leica DM4000B fluorescent microscope (1,000× magnification; Leica Microsystems, Wetzlar, Germany). The excitation and emission wavelengths were 480 and 635 nm, respectively. Cell density was calculated based on the method described in a previous publication (Ivanova et al., 2010). Active cells with intact plasma membrane structure fluoresced green, while dead cells with damaged plasma membrane structure appeared red. The cells were considered to have entered the VBNC state if a negative result was obtained only in the plate count but not in the live/dead BacLight™ Bacterial Viability staining for active bacteria.

### Scanning electron microscopy

Cell morphology of normal and induced VBNC cultures was observed using a scanning electron microscope. For scanning electron microscopy, bacterial cultures were centrifuged at 1,000× *g* at 4°C for 10 min, washed three times with 0.1 M phosphate-buffered saline, fixed in 2.5% glutaraldehyde for at least 2 h, washed three times with phosphate-buffered saline, and dehydrated through the 50, 70, 80, 90, and 100% ethanol series. Dehydrated cells were dried by carbon dioxide under a critical point, mounted, gold-coated, observed, and photographed using a scanning electron microscope (SU8010, Hitachi Ltd., Tokyo, Japan).

### Illumina high-throughput transcriptome sequencing

#### RNA extraction

Five samples of *L. paracasei* Zhang were collected at different stages of induction of the VBNC state (at 0, 3, 30, 180, and 210 days of induction), with three biological replicates for each condition. Total RNA was extracted using the LS1040 Eastep Super Total RNA Extraction Kit (Promega, Madison, WI, USA). The integrity of the extracted RNA was checked using an Agilent 2100 Bioanalyzer and agarose gel electrophoresis, and a NanoDrop2000 spectrophotometer was used to determine the purity and concentration of the extracted RNA. Qualified RNA samples (≥5 μg of total RNA amount; concentration ≥ 50 ng/μL; RNA integrity number ≥ 8.0) were used for cDNA library preparation.

#### cDNA library preparation for transcriptome sequencing

Ribosomal RNA (rRNA) was removed using the RiboCop rRNA Depletion Kit for mixed bacterial samples (Lexogen, Inc., Greenland, NH, USA). The RNA-seq transcriptome library was prepared using the Illumina^®^ Stranded mRNA Prep, Ligation (Illumina, Inc., San Diego, CA, USA) following the manufacturer's instructions. In brief, mRNAs were fragmented into short sequences of ~200 bases by adding a fragmentation buffer. Double-stranded cDNA was synthesized with random hexamer primers (Illumina Inc., San Diego, CA, USA). When the second strand cDNA was synthesized, dUTP was incorporated in place of dTTP. The synthesized cDNA was subjected to end-repair, phosphorylation, and “A” base addition. Paired-end RNA-seq libraries were sequenced on an Illumina HiSeq^TM^ 2000 platform at Shanghai Majorbio Bio-pharm Technology Co., Ltd., Shanghai, China.

#### Obtaining the clean read dataset and mapping to the reference genome

The Illumina platform converted the sequencing image signals to text signals by CASAVA base calling and storing the raw data in FASTQ format. Quality scores (such as base error rate, Q20, Q30, and GC content) were calculated. Quality trimming was performed by removing: the adaptor sequences from the reads; non-A, G, C, and T bases at the 5' end; low-sequencing quality reads scoring <Q20; reads containing up to 10% N; and those with a length of <25 bp after quality trimming. A clean/high-quality read dataset was obtained after such quality filtering.

The clean read dataset was mapped to the reference genome of *L. paracasei* Zhang (retrieved from http://www.ncbi.nlm.nih.gov/nuccore/CP001084) using the Burrows-Wheeler method by Bowtie2 (http://bowtie-bio.sourceforge.net/bowtie2/index.shtml). Mismatches of no more than five bases were allowed in the alignment.

#### Analysis of differential gene expression between samples

Differential gene expression in induced VBNC cells was identified by comparing the gene dataset of cells at different stages of induction with normal cells (collected at 0 days of induction). Raw counts were standardized using the trimmed mean of M values (TMM) normalization method (Robinson and Oshlack, 2010), and differential gene expression between groups was analyzed using DEGseq in the R package (Wang et al., 2009). Differentially expressed genes were identified based on a threshold of *P* (adjusted for multiple testing) <0.05 and fold change (FC) ≥ 2. Identified DEGs were subjected to the GO enrichment analysis (with the GO seq-based Wallenius non-central hypergeometric distribution) and the KEGG pathway enrichment analysis (Nguyen Thanh et al., 2014).

### Statistical analysis

Data were expressed as mean ± standard deviation (in triplicate). Excel and Origin 9.1 were used for data processing and analysis. Significant differences between groups were evaluated using *t*-tests with a cutoff level of *P* < 0.05.

## Results

### Prolonged incubation in a cold and acidic environment induced VBNC state

**Figure 1:**
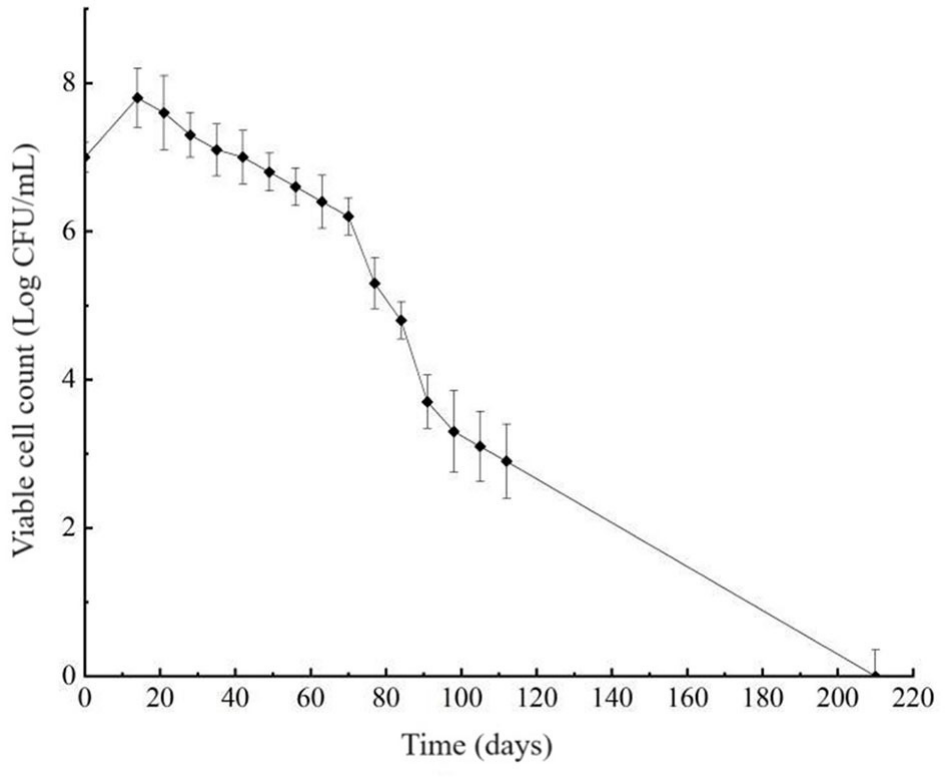
Induction curve of viable but non-culturable cells of *Lacticaseibacillus paracasei* Zhang. The plot shows the bacterial plate count against the time of induction. Cells were incubated in the de Man, Rogosa and Sharpe broth in a low temperature and acidic environment (4°C, pH 3.8) to induce the viable but non-culturable state.

The viability of *L. paracasei* Zhang cells was assessed weekly, showing a decline in cultivability as the incubation in the cold and acidic environment continued. After 210 days of induction for the VBNC state, the plate count dropped below 0.1 CFU/mL (Figure 1), suggesting a potential transition of cells to the VBNC state.

### Confirming the VBNC state by cell activity detection

**Figure 2:**
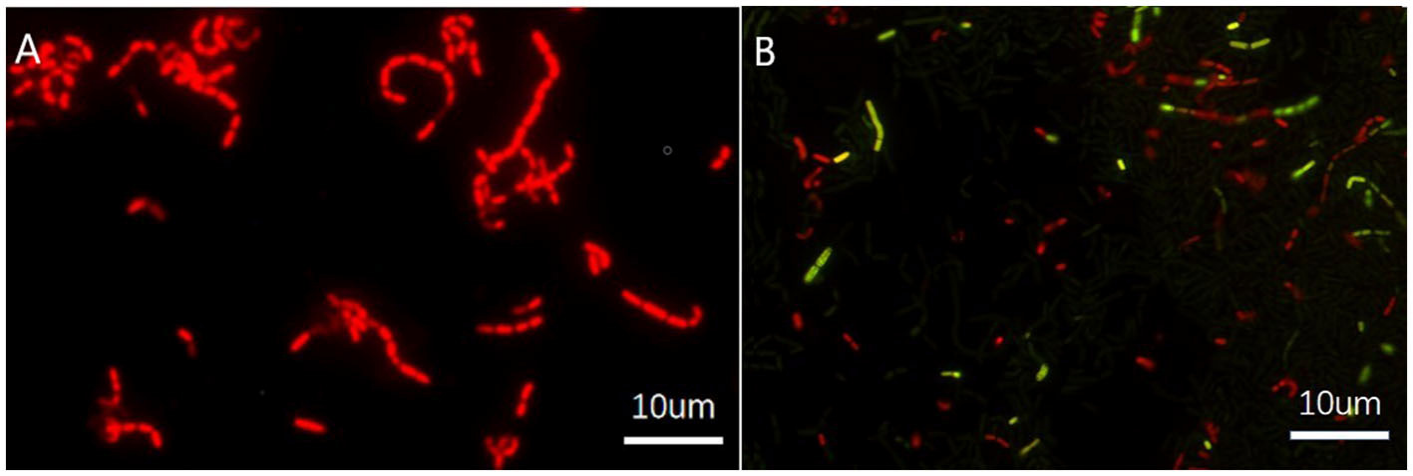
Fluorescence micrographs of *Lacticaseibacillus paracasei* Zhang. **(A)** Dead cells; **(B)** a culture of viable and non-culturable cells, stained with the Live/dead BacLight™ Bacterial Viability Kit (with two dyes, namely propidium iodide and SYTO 9). In micrograph **(B)**, viable and potentially active cells fluoresce green, while red fluorescing cells are dead bacteria. Although viable and non-culturable cells are alive, they are not detected by the traditional plate count method.

The transition to the VBNC state was further confirmed by staining for cell activity with the Live/dead BacLight test kit™ (based on propidium iodide and SYTO 9 co-staining). Both VBNC and dead cells would return negative results using the traditional plate count method. However, cells that appeared red were dead bacteria (Figure 2A), while those fluoresced green were viable and active (Figure 2B).

### Ultramorphology of VBNC cells of *L. paracasei* zhang

**Figure 3:**
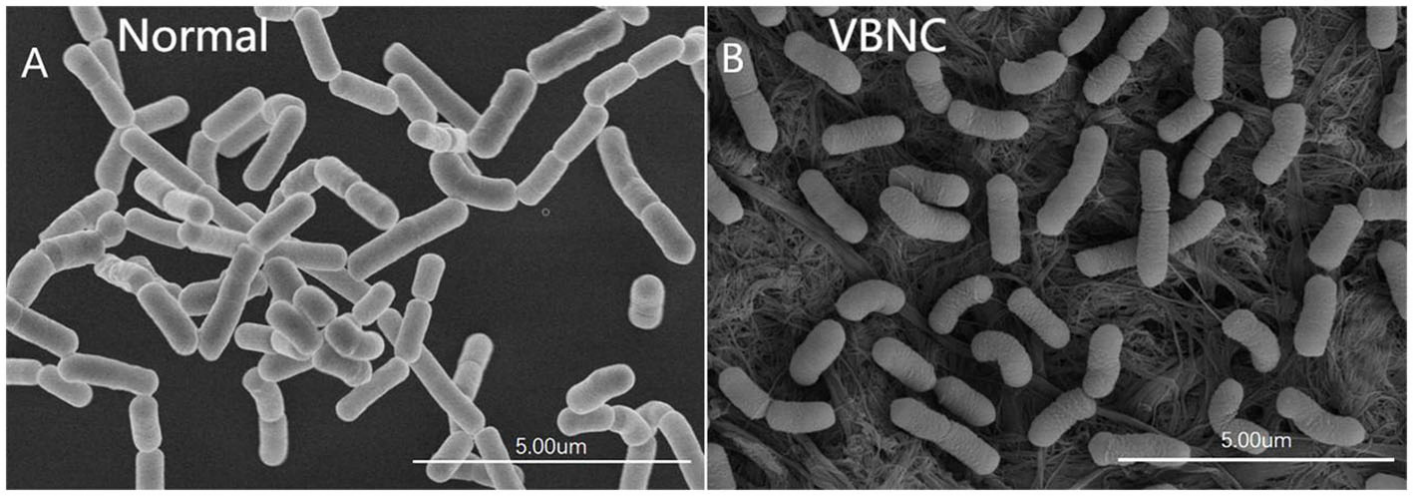
Scanning electron micrographs of **(A)** normal and **(B)** viable but non-culturable (VBNC) cells of *Lacticaseibacillus paracasei* Zhang.

The scanning electron micrographs show the typical ultramorphology of normal and VBNC *L. paracasei* Zhang cells (Figures 3A, B, respectively). Normal *L. paracasei* Zhang cells appeared as medium and short rods; VBNC cells were smaller and had a rough and slightly wrinkled surface.

### Quality of the extracted RNA

The extracted total RNA was quality-checked using agarose gel electrophoresis (Supplementary Figure S1). On the agarose gel, samples from earlier stages of VBNC state induction appeared as sharp bands, suggesting intact RNA preparation. As expected, the 23S bands were brighter than the 16S bands. Samples from 30 days before entering VBNC state and VBNC cells appeared as blurred smears, indicating some RNA degradation, suggesting that the RNA of VBNC cells might be more vulnerable to degradation during the sample processing and RNA extraction procedures.

The Agilent 2100 Biochip Analyzer was used to estimate the RNA concentration and purity of the extracted RNA samples (Supplementary Table S1). The RNA integrity number, concentration, and quantity of all samples were >8.0, ≥50 ng/μL, and ≥5 μg, respectively. All samples met the RNA quality criteria for further processing.

### Raw sequencing data and its quality control

The raw sequence data were analyzed, meeting the quality requirements of Q20 and Q30 scores of >90; a normal GC content ranging between 35 and 65%; and an acceptable rRNA contamination level of <8% (Supplementary Table S2). Thus, the quality of the generated raw data was acceptable.

Since the raw sequence dataset still contained linker sequences, high N-ratio sequences (representing fuzzy bases), and short-length reads, which would affect assembly quality and bioinformatics analysis accuracy, quality trimming was performed to generate a clean/high-quality sequence dataset; its sequencing statistical data are shown in Supplementary Table S3. The raw sequencing data were deposited at the NCBI Sequence Read Archive under the BioProject ID PRJNA989414.

### Mapping the clean dataset against the reference genome

The high-quality sequence dataset was compared against the whole *L. paracasei* Zhang genome using the Burrows-Wheeler method, yielding a mapping ratio of 89.95–97.38% (Supplementary Table S4), which exceeded the quality requirement of 65%. Thus, the quality trimmed sequence dataset was suitable for subsequent data analysis.

### Correlation between samples of different stages of VNBC state induction

**Figure 4:**
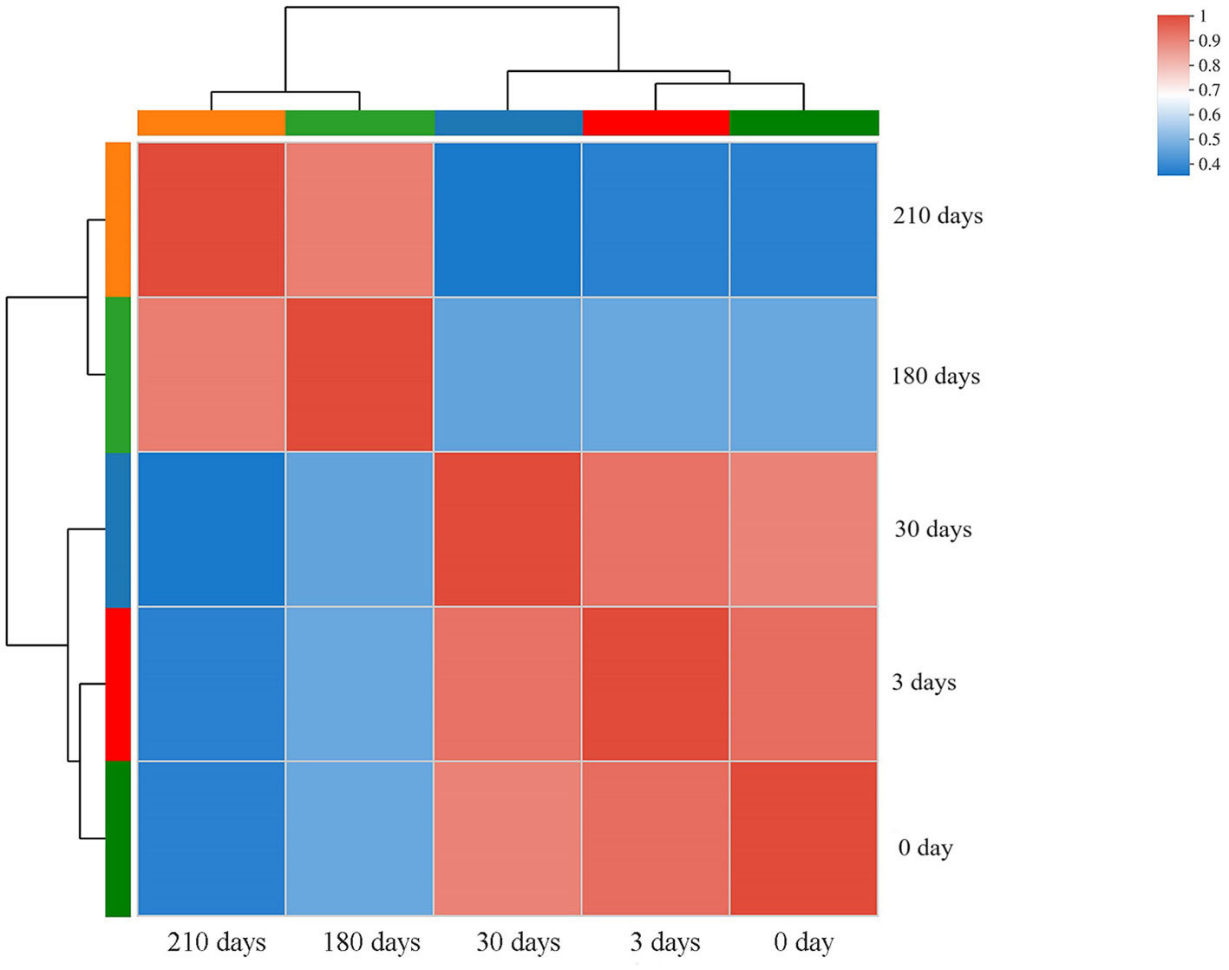
Correlation heatmap with hierarchical clustering constructed with different sample data subsets. The color scale represents the degree of correlation between sample pairs. Samples: 0, 3, 30, 180, and 210 days of induction for the viable but non-culturable state.

A correlation heatmap with hierarchical clustering was then constructed to illustrate the closeness between transcriptomic data subsets of samples collected at different VBNC induction stages. Two correlation clusters were observed: samples collected after 0, 3, and 30 days and samples collected after 180 days (30 days before VBNC entry) and 210 days of VBNC state induction (Figure 4). These results suggest that gradual changes occurred during the VBNC induction process, making it meaningful to compare gene expression between cultures at early and late stages of VBNC state transition.

### Changes in the transcriptomic profile and its function during the VBNC state transition

**Figure 5:**
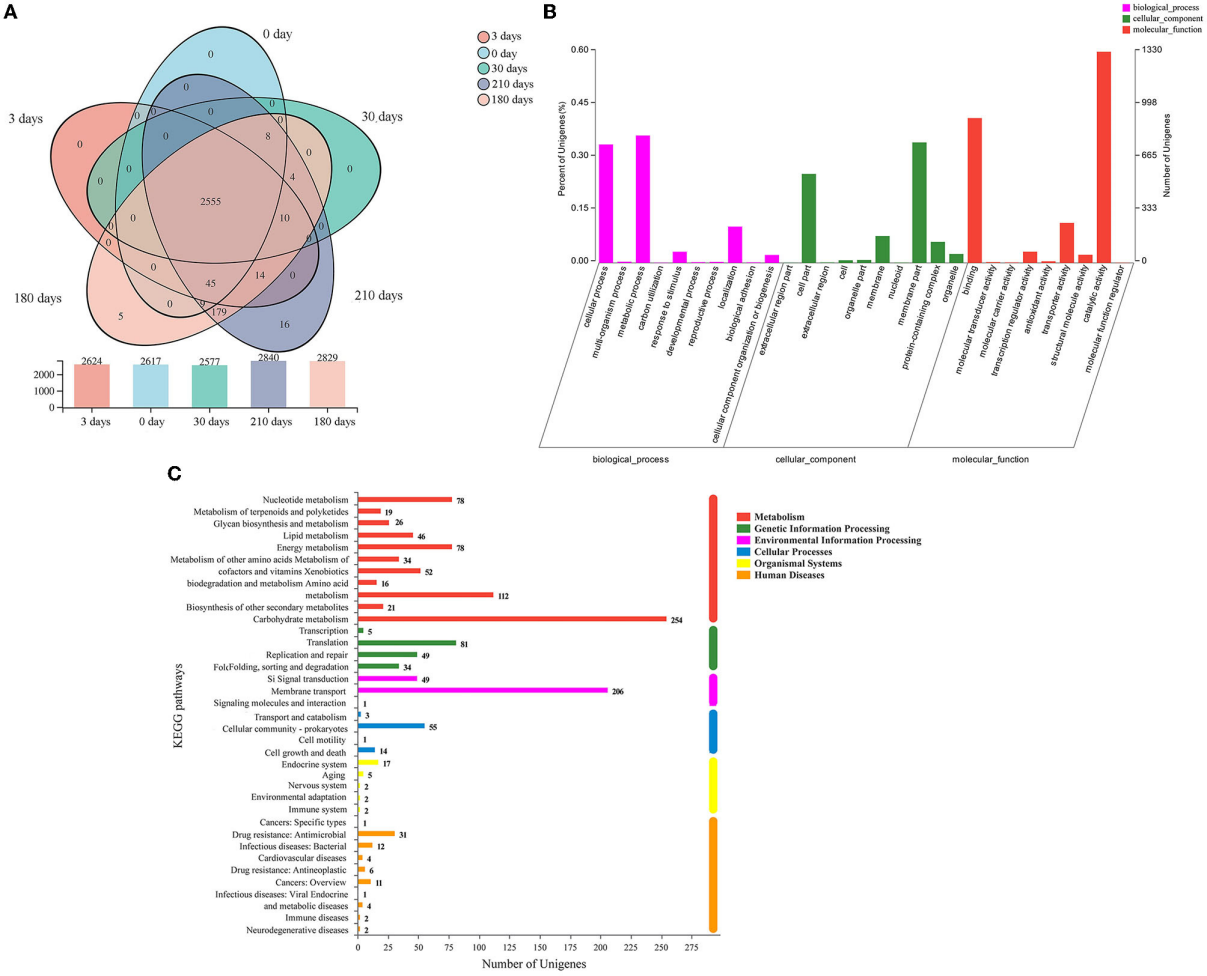
**(A)** Venn diagram of co-expressed and stage-specific genes in different samples collected at 0, 3, 30, 180, and 210 days of induction for the viable but non-culturable state. The bar chart below shows the number of genes identified in the respective sample. **(B)** Gene Ontology (GO) annotation results. The identified genes were categorized into the three branches of GO functions, namely, biological process, cellular component, and molecular function. **(C)** Results of pathway-level Kyoto Encyclopedia of Genes and Genomes (KEGG) database annotation. The ordinate is the KEGG pathway, and the abscissa is the number of genes annotated to the corresponding pathway.

The distribution of co-expressed and stage-specific genes is shown in the Venn diagram (Figure 5A). During the VBNC state induction, the number of co-expressed genes was 2,600 between 0 and 3 days, 2,563 between 0 and 30 days, 2,617 between 0 and 180 days, and 2,617 between 0 and 210 days. Five and 16 uniquely expressed genes were identified in samples induced for 180 days and 210 days, respectively (Figure 5A). To identify VBNC-stage-specific functional changes, the datasets were annotated using the GO database (Figure 5B). The identified genes were classified into three main GO functions: biological processes (BP; mainly cellular process and metabolic process), cellular components (CC; mainly membrane part and cell part), and molecular function (MF; mainly catalytic activity and binding). The KEGG database integrates genome, chemistry, and system function information, annotating genes based on fully sequenced genomes with system functions at the cell, species, and ecosystem levels. Our dataset was also annotated using the KEGG database; the top three gene categories were carbohydrate metabolism, membrane transport, and metabolism (Figure 5C).

A previous study found that low temperature could induce *Escherichia coli* O157:H7 to enter the VBNC state, resulting in increased expression in genes involved in ion transport, protein synthesis, and protein transmembrane transport activity, which is likely related to the need for protein synthesis aiming to maintain basal functional metabolism and vital activity during the VBNC state (Zhong and Zhao, 2019). The DEGs in VBNC cells were further analyzed using the GO and KEGG pathway enrichment analyses.

### GO and KEGG enrichment analyses of DEGs

**Figure 6:**
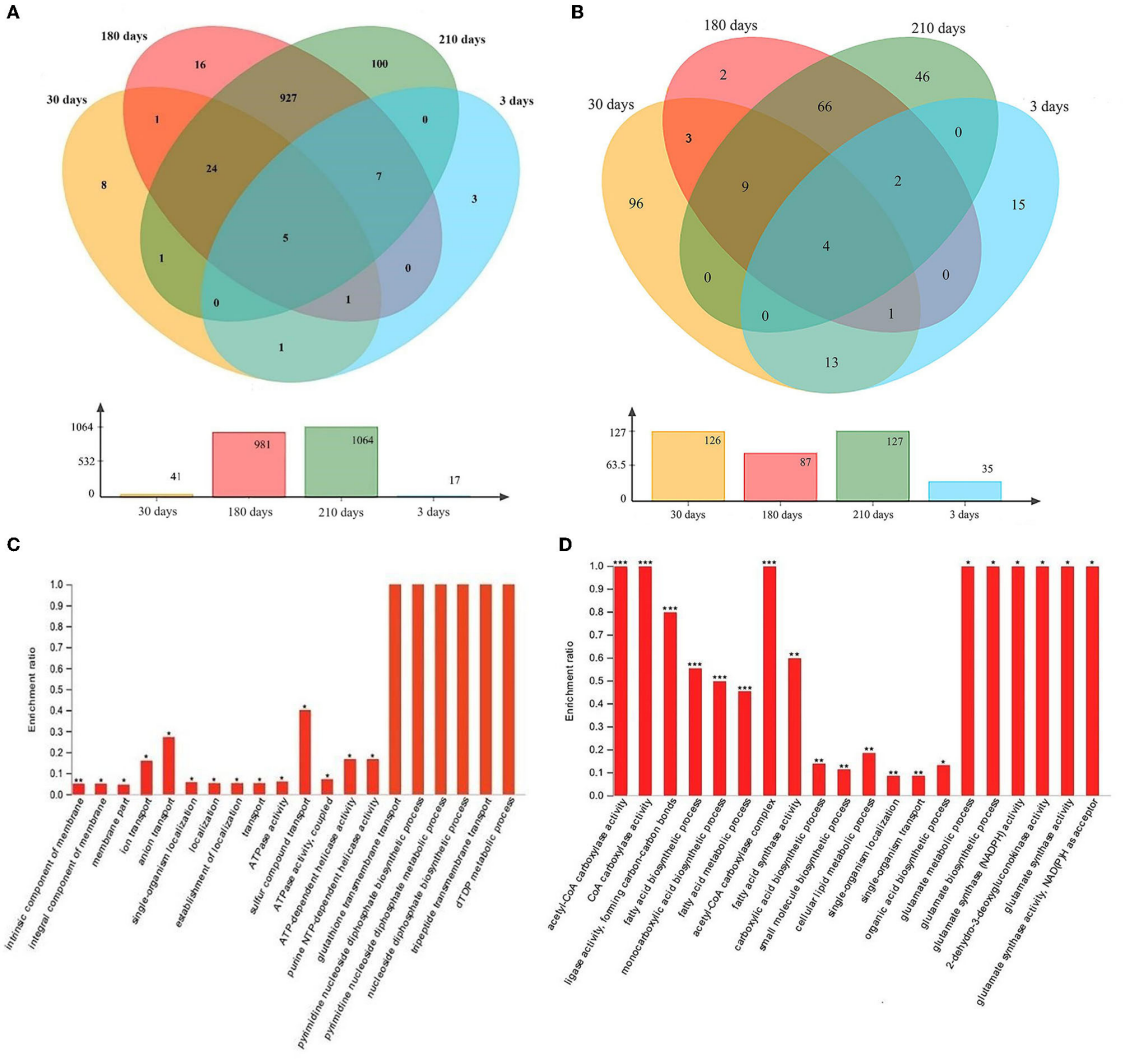
Venn diagram showing at least 2-fold **(A)** upregulated and **(B)** downregulated differentially expressed genes during the process of induction for the viable but non-culturable (VBNC) state. Samples were collected after 3, 30, 180, and 210 days of induction for the VBNC state. The bar charts below the Venn diagrams show the number of differentially expressed genes at each specific time point. Significantly **(C)** upregulated and **(D)** downregulated Gene Ontology functions in VBNC cells (collected after 210 days of induction) compared to uninduced cells (collected after 0 days of induction) are shown in the bar charts. *P-adjusted < 0.05; **P-adjusted < 0.01; ***P-adjusted < 0.001; Fisher's exact test, adjusted for multiple testing.

In total, 3, 8, 16, and 100 upregulated DEGs (at least 2-fold upregulation) were identified in samples collected after 3, 30, 180, and 210 days of induction for the VBNC state compared to normal cells (0 days of induction; Figure 6A). Five genes were consistently upregulated at all four time points following the induction for the VBNC state (Figure 6A). A total of 15, 96, 2, and 46 downregulated DEGs (at least 2-fold downregulation) were identified in samples collected after 3, 30, 180, and 210 days of induction for the VBNC state compared to normal cells (0 days of induction; Figure 6B). Four genes were consistently downregulated at all four time points following the induction of the VBNC state (Figure 6B). These DEGs may be associated with the transition to the VBNC state. However, further GO enrichment analysis failed to identify most DEGs as significantly enriched (*P*-adjust < 0.05; Fisher's exact test), except those from 210-day samples, despite a 2-fold upregulation compared to healthy cells. The enriched functions mainly included glutathione transmembrane transport, pyrimidine diphosphate, pyrimidine diphosphate, tripeptide, and dTDP metabolism (Figure 6C). The significantly deprived function (*P*-adjust < 0.05; Fisher's exact test) mainly belonged to acetyl-CoA carboxylase, glutamate metabolism process, glutamate biosynthesis process, glutamate synthase (NADPH) activity, 2-dehydro-3-deoxyglucokinase, glutamate synthase activity, glutamate synthase activity, and NADPH as receptors (Figure 6D).

To identify KEGG pathways associated with the VBNC state transition, we conducted a KEGG enrichment analysis, identifying seven enriched pathways from the upregulated DEGs, such as fructose and mannose metabolism, amino sugar and nucleotide sugar metabolism, starch and sucrose metabolism, and amino sugar and nucleotide sugar metabolism (Supplementary Table S5), and 24 deprived KEGG pathways, such as PTS, fatty acid synthesis, and quorum sensing, from the downregulated DEGs (Supplementary Table S6).

### GO and KEGG analyses of consistently up/downregulated genes during VBNC state transition

**Figure 7:**
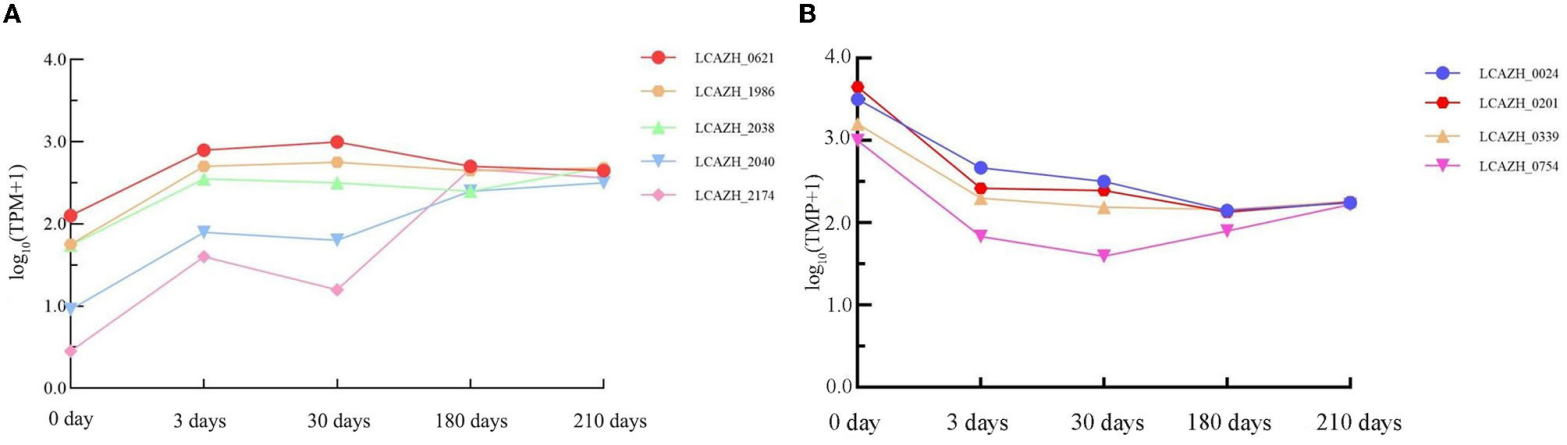
Changes in transcription frequency of genes showing consistent **(A)** upregulation and **(B)** downregulation in induced cells compared to healthy cells during the process of viable but non-culturable state transition. Samples were collected after 0, 3, 30, 180, and 210 days of induction for the viable but non-culturable state. TPM, transcripts per million.

Five genes were consistently upregulated during the VBNC state transition compared to uninduced cells, but only two were significantly enriched in the GO and KEGG enrichment pathway analyses, namely, LCAZH_0621 and LCAZH_2040 (Figure 7A; Supplementary Table S7).

LCAZH_0621 encodes an A/G-specific adenine glycosylase; it was upregulated 2.7-fold after 3 days, 2.7-fold after 30 days, 2.6-fold after 180 days, and 2.6-fold after 210 days of VBNC state induction. The GO enrichment pathways of LCAZH_0621 are GO: 0006284 and GO: 0003677 (representing BP: base excision repair; MF: DNA binding, respectively), which are involved in map03410 (base excision repair). The continuous upregulated LCAZH_0621 expression suggests its crucial role in repairing DNA damage upon prolonged exposure to a low temperature and pH environment.

LCAZH_2040 encodes a PTS fiber biospecific transporter IIC subunit; it was upregulated 1.9-fold after 3 days, 1.8-fold after 30 days, 2.3-fold after 180 days, and 2.4-fold after 210 days of VBNC state induction. It is involved in GO: 0009401; GO: 0016021; and GO: 0005886 (representing BP: phosphoenolpyruvate-dependent sugar phosphotransferase system; CC: membrane; and CC: plasma membrane, respectively). It participates in map02060 (PTS) and map00500 (starch and sucrose metabolism). A previous report found that PTSs play roles in carbohydrate transport and regulation of metabolism; *L. paracasei* Zhang showed a substantially higher PTS activity when grown in normal conditions compared to a cold and acidic environment (Zhang et al., 2017). The current observation and the results reported by Zhang et al. (2017) suggest an increase in PTS gene expression may enhance the stress resistance of this bacterial strain.

LCAZH_1986, LCAZH_2038, and LCZH_2174 encode an arsenate reductase and two hypothetical proteins, respectively. The biological roles of these genes in the VBNC state await further elucidation.

Four genes were consistently downregulated during the VBNC state transition compared to uninduced cells (Figure 7B, Supplementary Table S8). These four genes encode two oligopeptide ABC transporter periplasmic proteins (LCAZH_0201 and LCAZH_0339), the catabolite control protein A (LCAZH_0754), and a surface antigen (LCAZH_0024), respectively.

The expression of LCAZH_0201 was downregulated 2.3-fold after 3 days, 2.1-fold after 30 days, 2.3-fold after 180 days, and 2.4-fold after 210 days of VBNC state induction. The expression of LCAZH_0339 was downregulated 1.9-fold after 3 days, 1.6-fold after 30 days, 2.15-fold after 180 days, and 2.28-fold after 210 days of VBNC state induction. The GO pathways of LCAZH_0201 and LCAZH_0339 are GO: 0055085 and GO:0043190 (representing BP: transmembrane transport; CC: ATP-binding cassette (ABC) transporter complex, respectively). These GO terms and pathways are involved in map02024, map02010, and map01501 (assigned as quorum sensing, ABC transporters, and β-lactam resistance, respectively). The decreased expression in LCAZH_0201 and LCAZH_0339 during the transition to the VBNC state indicates that cells could remain viable but maintain low activity and ATP levels, potentially extending their survival time in unfavorable environments.

LCAZH_0754 was downregulated 2.37-fold after 3 days, 2.36-fold after 30 days, 2.3-fold after 180 days, and 2.4-fold after 210 days of VBNC state induction. The GO pathways of LCAZH_0754 are GO: 0006355; GO: 0006351; GO: 0045892; GO: 0032993; GO: 0003677; GO: 0000976; GO: 0001217 (representing BP: regulation of DNA-templated transcription; BP: DNA-templated transcription; BP: negative regulation of DNA-templated transcription; CC: protein–DNA complex; MF: DNA binding; MF: transcription cis-regulatory region binding; MF: DNA binding transcription repressor activity, respectively). These results may suggest that *L. paracasei* Zhang can regulate gene transcription by reducing the expression of catabolite control protein A to strive for survival in a low temperature and acidity environment.

The expression level of LCAZH_0024 (surface antigen) was downregulated 2.6-fold after 3 days, 2.5-fold after 30 days, 2.3-fold after 180 days, and 2.3-fold after 210 days of VBNC state induction. Its gene product is involved in GO: 0016787 (MF: hydrolase activity), and the reason for its downregulation remains to be investigated.

## Discussion

*Lacticaseibacillus paracasei* Zhang is a widely used starter culture and probiotic in the food and health food industry. The bacteria transit to the VBNC state when facing adverse environments, which can minimize cell activity and growth and reduce the number and density of viable bacteria, hindering the development of high-density fermentation technology and the manufacturing of highly effective probiotic products. Limited research exists on the VBNC state of probiotic bacteria, and its formation mechanism remains unclear. Thus, this study investigated the transcriptomic changes of *L. paracasei* Zhang in transition to the VBNC state.

Our research results confirmed that *L. paracasei* Zhang could enter the VBNC state after 210 days of incubation in an MRS medium at pH 3.8 and 4°C. The cultivability of VBNC *L. paracasei* Zhang cells could be restored when grown in MRS medium (pH 6.8), suggesting that lifting the acidic stress promotes the exit of the VBNC state in this bacterial strain. It is known that altering environmental factors (such as removal of external stress, supplementation with nutrients, peroxidase, or other resuscitation-promoting factors) may restore cell growth of VBNC cells, but the conditions for recovering VBNC cells may be specific to bacterial species (Pan and Ren, 2022). For example, it was found that *Staphylococcus aureus* could be induced by starving the cells and maintaining them in natural seawater at 4°C, and raising the temperature from 4 to 22°C partially recovered cultivability (Masmoudi et al., 2010). Two strains of *Arcobacter butzleri* became non-culturable in seawater microcosms at 4°C and at room temperature, and the cells could be resuscitated 9 days after nutrient addition (Fera et al., 2008). Few studies have been performed on VBNC lactic acid bacteria. A previous study found that lactic acid bacteria entered the VBNC state during wine storage when sulfur dioxide was added. Despite being no longer culturable, these bacteria still maintained membrane integrity and enzymatic activity. The study highlights the need to understand conditions for reversing and recovering the cultivability of VBNC lactic acid bacteria (Millet and Lonvaud-Funel, 2000).

An interesting observation of the ultramorphology of VBNC *L. paracasei* Zhang is the reduced cell size with a wrinkled and rough surface. Similar cell size reduction was reported by Millet and Lonvaud-Funel (2000), observing that filtrating wine stored in barrels through a 0.45-mm membrane failed to remove the VBNC bacteria, suggesting that bacteria shrunk in the VBNC state. These cell morphological changes might be part of the stress-protective response of cells, which has also been reported as a stress-related response in other bacteria (Zhong and Zhao, 2019).

This study utilized transcriptomic analysis to investigate the alterations in gene expression of *L. paracasei* Zhang at various time points during VBNC induction. Hierarchical clustering of the transcriptomic data subsets revealed that the gene expression profile of the early time points (0, 3, and 30 days) formed a different cluster from that of cells sampled at later time points of VBNC induction (180 and 210 days). Based on the results of cultivability and cell activity staining, it was confirmed that the cells entered the VBNC state between these two later time points. Thus, the transition to the VBNC state was accompanied by a shift of the cell transcriptome. Then, we screened our dataset to identify DEGs associated with the VBNC state transition in *L. paracasei* Zhang, revealing nine genes (five upregulated and four downregulated) that exhibited consistent up/downregulation during the process.

The upregulated genes, LCAZH_0621 and LCAZH_2040, encode the A/G-specific adenine glycosylase and the PTS system fiber biospecific transporter IIC subunit, respectively; these two genes participate in base repair and membrane translocation of substrates. Increased expression of LCAZH_0621 likely enhances the function of base excision repair and DNA binding, thereby elevating the stress resistance of the cells (Völzing and Brynildsen, 2015). Differential expression of the PTS system may help *L. paracasei* Zhang acquire and assimilate carbon sources other than glucose to maintain survival (Su et al., 2016). In contrast, LCAZH_0754 exhibited a consistent downregulation trend. It encodes catabolite control protein A, which is an inhibitor or activator of gene transcription in low-GC Gram-positive bacteria, responsible for regulating the biological metabolism of *Streptococcus bovis* (Jin et al., 2021). The downregulation of LCAZH_0754 might be associated with the quiescency of VBNC *L. paracasei* Zhang cells. The functions of other consistently differentially regulated genes in sustaining the VBNC state are less clear and remain to be determined in future studies.

A limitation of the current study is that reverse genetic experiments were not performed, which would be useful to reveal and validate the roles of these consistent DEGs in VBNC bacteria. Nevertheless, data generated in this study provide a starting point for in-depth analysis of the biology of survival of a probiotic in the VBNC state.

## Conclusion

The study found that *L. paracasei* Zhang, a dairy-associated probiotic strain, can enter the VBNC state through long-term maintenance in a cold and acidic condition. Global transcriptomics identified upregulated (such as base excision repair, PTS, starch, and sucrose metabolic pathways) and downregulated (such as ABC transporter and catabolite control protein A) DEGs and pathways in bacterial cells at different stages of VBNC induction. We hypothesize that the regulation of these genes is associated with cell entry into the VBNC state, where cells must adopt a low metabolism mode with minimal cell activity and maximal substrate utilization efficiency to maximize their survival. Our study provides preliminary data on the molecular modulation of *L. paracasei* Zhang in the transition to the intriguing VBNC state, which is of interest to microbiologists and dairy scientists.

## Data availability statement

The datasets presented in this study can be found in online repositories. The names of the repository/repositories and accession number(s) can be found in the article/Supplementary material.

## Author contributions

QB: Conceptualization, Funding acquisition, Methodology, Project administration, Resources, Validation, Writing – original draft, Writing – review & editing. XM: Conceptualization, Data curation, Formal analysis, Investigation, Validation, Writing – original draft, Writing – review & editing. XB: Writing – review & editing, Software. JP: Methodology, Writing – review & editing, Visualization. LD: Writing – review & editing, Methodology. HW: Software, Writing – review & editing. YC: Funding acquisition, Supervision, Writing – review & editing. L-YK: Funding acquisition, Supervision, Writing – review & editing.
