# Supplementary material for: Transcriptomic analysis of Lacticaseibacillus paracasei Zhang in transition to the viable but non-culturable state by RNA sequencing
Source: Front Microbiol. 2023 Dec 21;14:1280350. doi: 10.3389/fmicb.2023.1280350 (PMC10768001; doi:10.3389/fmicb.2023.1280350)
Supplement: Supplementary file 1 [file Data_Sheet_1.docx]

Supplementary Material

Transcriptomics Analysis of *Lacticaseibacillus paracasei* Zhang in Transition to the Viable but Nonculturable State by RNA-Sequencing

Qiuhua Bao ^1,2,3†^, Xuebo Ma ^1,2,3†^, Xiaoyu Bo ^1,2,3^, Jing Pang ^1,2,3^, Lixia Dai ^1,2,3^,

Huiying Wang ^1,2,3^, Yongfu Chen ^1,2,3*^, Lai-Yu Kwok ^1,2,3*^

^1^ Key Laboratory of Dairy Biotechnology and Engineering, Ministry of Education, Inner Mongolia Agricultural University, Hohhot 010018, China

^2^ Key Laboratory of Dairy Products Processing, Ministry of Agriculture and Rural Affairs, Inner Mongolia Agricultural University, Hohhot 010018, China

^3^ Inner Mongolia Key Laboratory of Dairy Biotechnology and Engineering, Inner Mongolia Agricultural University, Hohhot 010018, China

^†^ These authors contributed equally to this work and share the first authorship

*** Correspondence:**Yongfu Chen, Key Laboratory of Dairy Biotechnology and Engineering, Education Ministry of P. R. China, 306 Zhaowuda Street, Hohhot, China, 010018. Tel: 86-471-4300591, Fax: 86-471-4305357, E-mail: nmgyfchen@126.com

Lai-Yu Kwok, Key Laboratory of Dairy Biotechnology and Engineering, Education Ministry of P. R. China, 306 Zhaowuda Street, Hohhot, China, 010018. Tel: 86-471-4300591, Fax: 86-471-4305357, E-mail: kwok_ly@yahoo.com

# Supplementary Figures and Tables

## Supplementary Figure


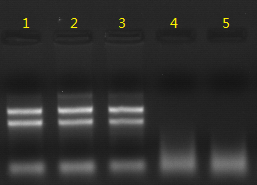


**Supplementary Figure 1.** Evaluation of total RNA quality by agarose gel electrophoresis. Lanes 1 to 5 correspond to the extracted RNA from samples collected after 0, 3, 30, 180 (30 days before entering the viable but nonculturable [VBNC] state), and 210 days (cells entered into the VBNC state) of induction for the VBNC state, respectively.

**
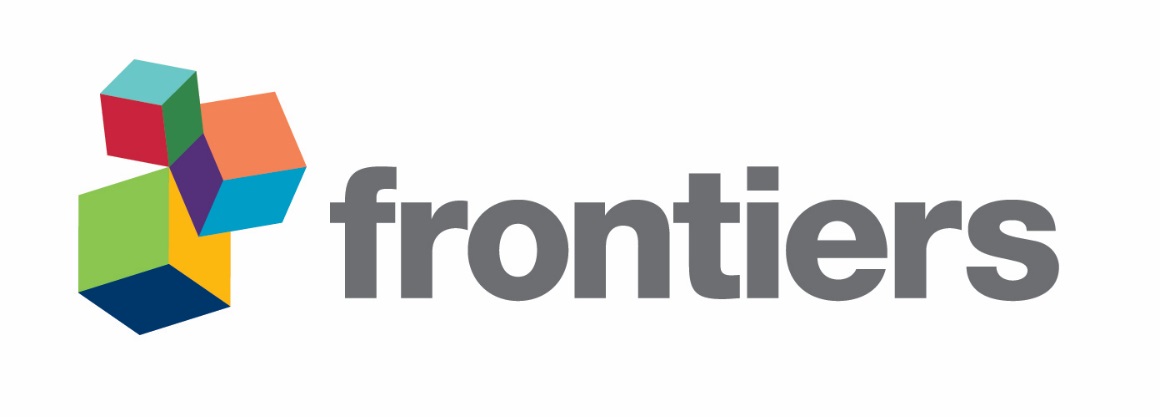
**

**Supplementary Tables**

**Table S1** Evaluation of RNA integrity of samples by Agilent Technologies 2100

| **Sample** | **Concentration (ng/ul)** | **Total RNA amount (ug)** | **OD_260/280_** | **OD_260/230_** | **RNA integrity number** |
| --- | --- | --- | --- | --- | --- |
| 0 day | 399.3 | 10.4 | 2.11 | 2.24 | 9.1 |
| 3 days | 828.4 | 39.8 | 2.18 | 2.21 | 8.5 |
| 30 days | 258.6 | 6.5 | 2.11 | 2.18 | 9.5 |
| 180 days | 996.7 | 84.7 | 2.04 | 2.33 | 9.0 |
| 210 days | 560 | 25.2 | 1.93 | 1.36 | 8.8 |

Remark: Samples were collected after different durations of induction for the viable but unculturable state.

**Table S2** Summary statistics of raw sequence dataset

| **Sample** | **Number of sequences** | **Number of nucleotide bases** | **Base error rate (%)** | **Q20 (%)** | **Q30 (%)** | **GC content (%)** |
| --- | --- | --- | --- | --- | --- | --- |
| 0 day | 15062632 | 2259394800 | 0.01 | 98.21 | 95.51 | 47.62 |
| 3 days | 15286870 | 2293030500 | 0.0099 | 98.2 | 95.55 | 47.52 |
| 30 days | 13776908 | 2066536200 | 0.0099 | 98.26 | 95.65 | 47.63 |
| 180 days | 27359758 | 4103963700 | 0.0118 | 96.95 | 93.17 | 48.09 |
| 210 days | 29117486 | 4367622900 | 0.0121 | 96.71 | 92.74 | 47.86 |

Remarks: Samples were collected after different durations of induction for the viable but unculturable state. Q20 and Q30 represent the percentage of bases with a Phred value greater than 20 or 30 of the total bases, respectively.

**Table S3** Summary statistics of the high-quality sequence dataset

| **Sample** | **Number of sequences** | **Number of nucleotide bases** | **Base error rate (%)** | **Q20 (%)** | **Q30 (%)** | **GC content (%)** |
| --- | --- | --- | --- | --- | --- | --- |
| 0 day | 14910326 | 2204454032 | 0.0094 | 98.83 | 96.43 | 47.58 |
| 3 days | 15124350 | 2235203541 | 0.0093 | 98.86 | 96.52 | 47.47 |
| 30 days | 13635282 | 2016858242 | 0.0093 | 98.88 | 96.56 | 47.59 |
| 180 days | 26704448 | 3721765918 | 0.0101 | 98.49 | 95.48 | 47.56 |
| 210 days | 28326590 | 3948784464 | 0.0102 | 98.4 | 95.29 | 47.57 |

Remarks: Samples were collected after different durations of induction for the viable but unculturable state. Q20 and Q30 represent the percentage of bases with a Phred value greater than 20 or 30 of the total bases, respectively.

**Table S4** Mapping ratio statistics

| **Sample** | **Number of mapped/total sequences of the data subset** | **Mapping ratio (%)** |
| --- | --- | --- |
| 0 day | 14519076/14910326 | 97.38 |
| 3 days | 14700522/15124350 | 97.20 |
| 30 days | 13272288/13635282 | 97.34 |
| 180 days | 24020714/26704448 | 89.95 |
| 210 days | 25545352/28326590 | 90.18 |

Remark: Samples were collected after different durations of induction for the viable but unculturable state.

**Table S5** Enriched KEGG pathways in cells in transition to the viable but unculturable state

| **Pathway ID** | **Pathway description** | **Number of DEGs detected/Number of genes in this pathway** | ***P*-value** |
| --- | --- | --- | --- |
| map00051 | Fructose and mannose metabolism | 1/1 | 0.0336 |
| map00520 | Amino sugar and nucleotide sugar metabolism | 1/1 | 0.0308 |
| map00500 | Starch and sucrose metabolism | 2/3 | 0.0032 |
| map00520 | Amino sugar and nucleotide sugar metabolism | 2/3 | 0.0027 |
| map04112 | Cell cycle - *Caulobacter* | 1/3 | 0.0250 |
| map00401 | Novobiocin biosynthesis | 1/34 | 0.0470 |
| map00960 | Tropane, piperidine and pyridine alkaloid biosynthesis | 1/34 | 0.0470 |

Remarks: KEGG means Kyoto Encyclopedia of Genes and Genomes; DEG means differentially expressed gene.

**Table S6** Deprived KEGG pathways in cells in transition to the viable but unculturable state

| **Pathway ID** | **Pathway description** | **Number of DEGs detected/Number of genes in this pathway** | ***P*-value** |
| --- | --- | --- | --- |
| map02060 | Phosphotransferase system (PTS) | 6/12 | 0 |
| map00052 | Galactose metabolism | 4/12 | 0.0003 |
| map00051 | Fructose and mannose metabolism | 4/12 | 0.0005 |
| map05111 | Biofilm formation - *Vibrio cholerae* | 1/12 | 0.0250 |
| map02026 | Biofilm formation | 1/12 | 0.0494 |
| map00061 | Fatty acid biosynthesis | 8/63 | 0 |
| map02024 | Quorum sensing | 10/63 | 0 |
| map00720 | Carbon fixation pathways in prokaryotes | 4/63 | 0.0032 |
| map02010 | ABC transporters | 11/63 | 0.0047 |
| map00640 | Propanoate metabolism | 4/63 | 0.0081 |
| map00910 | Nitrogen metabolism | 2/63 | 0.0256 |
| map01055 | Biosynthesis of vancomycin group antibiotics | 1/63 | 0.0441 |
| map01501 | beta-Lactam resistance | 3/63 | 0.0414 |
| map00040 | Pentose and glucuronate interconversions | 2/63 | 0.0348 |
| map00780 | Biotin metabolism | 2/63 | 0.0348 |
| map03010 | Ribosome | 11/31 | 0 |
| map04212 | Longevity regulating pathway - worm | 2/31 | 0.0027 |
| map03020 | RNA polymerase | 2/31 | 0.0044 |
| map00195 | Photosynthesis | 2/31 | 0.0117 |
| map04940 | Type I diabetes mellitus | 1/31 | 0.0217 |
| map04112 | Cell cycle - *Caulobacter* | 2/31 | 0.0262 |
| map00710 | Carbon fixation in photosynthetic organisms | 2/31 | 0.0452 |
| map05134 | Legionellosis | 1/31 | 0.0429 |
| map04141 | Protein processing in endoplasmic reticulum | 1/31 | 0.0429 |

Remarks: KEGG means Kyoto Encyclopedia of Genes and Genomes; DEG means differentially expressed gene.

**Table S7** Genes consistently up-regulated in transition to the viable but unculturable state

| **Gene ID** | **Gene description** | **GO ID** | **GO term** | **KEGG pathway ID** | **Pathway definition** |
| --- | --- | --- | --- | --- | --- |
| LCAZH_0621 | A/G specific adenine glycosylase | GO: 0006284  GO: 0003677 | BP: Base-excision repair  MF: DNA binding | map03410 | Base excision repair |
| LCAZH_1986 | Arsenate reductase | ------ | ------ | ------ | ------ |
| LCAZH_2038  LCAZH_2174 | Hypothetical protein | ------ | ------ | ------ | ------ |
| LCAZH_2040 | PTS Systemic fiber specific transporter IIC | GO: 0009401  GO: 0016021  GO: 0005886 | BP: Phosphoenolpyruvate-dependent glucose phosphotransferase system  CC: Membrane  CC: Plasma membrane | map02060  map00500 | Phosphotransferase system  Starch and sucrose metabolism |

Remarks: KEGG means Kyoto Encyclopedia of Genes and Genomes; GO means Gene Ontology; BP means biological processes; CC means cellular components; MF means molecular function.

**Table S8** Genes consistently down-regulated in transition to the viable but unculturable state

| **Gene ID** | **Gene description** | **GO ID** | **GO term** | **KEGG Pathway** | **Pathway definition** |
| --- | --- | --- | --- | --- | --- |
| LCAZH_0024 | Surface antigen | GO: 0016787 | MF: Hydrolase activity | ------ | ------ |
| LCAZH_0201  LCAZH_0339 | Oligopeptide ABC transporter periplasmic protein  Oligopeptide ABC transporter periplasmic protein | GO: 0055085  GO: 0043190 | BP: Transmembrane transport  CC: ATP-binding cassette (ABC) transporter complex | map02024  map02010  map01501 | Quorum sensing  ABC transporters  β-Lactam resistance |
| LCAZH_0754 | Catabolic control protein A | GO: 0006355  GO: 0006351  GO: 0045892  GO: 0032993  GO: 0003677  GO: 0000976  GO: 0001217 | BP: Regulation of DNA-templated transcription  BP: DNA-templated transcription  BP: Negative regulation of DNA templated transcription  CC: Protein-DNA complex  MF: DNA binding  MF: Transcription cis-regulatory region binding  MF: DNA-binding transcription repressor activity | ------ | ------ |

Remarks: KEGG means Kyoto Encyclopedia of Genes and Genomes; GO means Gene Ontology; BP means biological processes; CC means cellular components; MF means molecular function.
